# Extensively duplicated and transcriptionally active recent lateral gene transfer from a bacterial *Wolbachia* endosymbiont to its host filarial nematode *Brugia malayi*

**DOI:** 10.1186/1471-2164-14-639

**Published:** 2013-09-22

**Authors:** Panagiotis Ioannidis, Kelly L Johnston, David R Riley, Nikhil Kumar, James R White, Karen T Olarte, Sandra Ott, Luke J Tallon, Jeremy M Foster, Mark J Taylor, Julie C Dunning Hotopp

**Affiliations:** 1 Institute for Genome Sciences, University of Maryland School of Medicine, Baltimore, MD, USA; 2 Liverpool School of Tropical Medicine, Liverpool, UK; 3 New England Biolabs, Ipswich, MA, USA; 4 Department of Microbiology and Immunology, University of Maryland School of Medicine, Baltimore, MD, USA; 5 Current address: Department of Genetic Medicine and Development, University of Geneva Medical School, 1211 Geneva, Switzerland

## Abstract

**Background:**

Lymphatic filariasis is a neglected tropical disease afflicting more than 120 million people, while another 1.3 billion people are at risk of infection. The nematode worm *Brugia malayi* is one of the causative agents of the disease and exists in a mutualistic symbiosis with *Wolbachia* bacteria. Since extensive lateral gene transfer occurs frequently between *Wolbachia* and its hosts, we sought to measure the extent of such LGT in *B. malayi* by whole genome sequencing of *Wolbachia*-depleted worms.

**Results:**

A considerable fraction (at least 115.4-kbp, or 10.6%) of the 1.08-Mbp *Wolbachia w*Bm genome has been transferred to its nematode host and retains high levels of similarity, including 227 *w*Bm genes and gene fragments. Complete open reading frames were transferred for 32 of these genes, meaning they have the potential to produce functional proteins. Moreover, four transfers have evidence of life stage-specific regulation of transcription at levels similar to other nematode transcripts, strengthening the possibility that they are functional.

**Conclusions:**

There is extensive and ongoing transfer of *Wolbachia* DNA to the worm genome and some transfers are transcribed in a stage-specific manner at biologically relevant levels.

## Background

*Brugia malayi* (filarial nematode) is a causative agent of human lymphatic filariasis, a neglected tropical disease that results in elephantiasis and thus disability, handicap, and stigma. Over 120 million people have lymphatic filariasis, with another 1.3 billion people at risk of infection [1]. Transmission of the disease requires a mosquito vector, which ingests microfilariae from an infected human blood meal. The parasites develop into infective 3rd stage larvae (L3) inside the mosquito and are subsequently transmitted to another human during the next blood meal [1]. Efforts at combating the disease include mass drug administration to reduce the blood levels of microfilariae, the transmissible form. This scheme aims only at interrupting further transmission of the disease, as these drugs do not affect adult worms. Antibiotics kill all life stages by targeting the obligate mutualistic *Wolbachia* symbiosis, and thus can be used to treat lymphatic filariasis [2]. These *Wolbachia* endosymbionts are α-Proteobacteria found in all three of the causative agents of lymphatic filariasis, namely *Wuchereria bancrofti*, *B. malayi,* and *Brugia timori*[3].

During the original whole genome sequencing of *B. malayi* extensive levels of lateral gene transfer (LGT) were identified from its *Wolbachia* endosymbiont, *w*Bm [4]. LGT is the process whereby organisms acquire DNA from other organisms in the absence of sex. LGT from the *Wolbachia* genome to the nuclear genome of its eukaryotic hosts is widespread [5,6]. In a search of the sequence data archives, 20-30% of arthropods and nematodes have evidence for LGT from *Wolbachia*[4,6]. More remarkably, 80% of species containing *Wolbachia* had evidence of LGT [4]. Of the five species examined further, all of the LGTs examined were confirmed experimentally. Frequently, *Wolbachia* DNA is detected in the host genome [4,7-15], including transfers of >10% of the *Wolbachia* genome [4,9,14]. Such LGTs are called *nuwts* for *nu*clear W*olbachia t*ransfers following the established nomenclature for *numts* for *nu*clear *m*i*t*ochondrial DNA segments.

Most of the nuwts detected previously in *B. malayi* are degenerate [4], suggesting that there is no selective pressure to maintain their functionality. However, the methods used to assemble the *B. malayi* genome would favor the discovery of degenerate sequences. Since the endosymbiont is an obligate symbiont, the nematode genome and bacterial genome were sequenced simultaneously. Therefore, to assemble the *B. malayi* genome, reads that were >98% similar to *Wolbachia w*Bm over >90% of their length were removed from the assembly [4]. This leads to the removal of the most conserved sequences. In addition, regions adjacent to nuwts that were removed in the screen, as well as duplicated regions, are unlikely to be well resolved in the assembly. Therefore, we sought to quantify the size and number of nuwts in the filarial nematode *B. malayi* genome that arise from its bacterial endosymbiont, *Wolbachia* sp. *w*Bm. Using genome sequencing of *Wolbachia*-depleted worms, we obtained the full list of nuwts in *B. malayi*. Intriguingly, this list includes several full-length *Wolbachia* genes with the potential to be functional that are also shown to generate stage-specific transcripts.

## Results

### *Wolbachia* depletion of *Brugia malayi* worms and DNA sequencing

Since *B. malayi* has a mutualistic symbiotic relationship with *Wolbachia* strain *w*Bm, such that neither symbiotic partner can survive without the other, the *B. malayi* genome cannot be sequenced without also sequencing the *Wolbachia* genome. This complicates identification of nuwts when compared to detecting *Wolbachia*-host LGT in naturally *Wolbachia*-free nematodes [11,16] or insects that can be cured of their *Wolbachia* infection with antibiotics [4,12]. To overcome this, a *Wolbachia*-depletion approach was undertaken in order to examine worms with low, but not immediately lethal, *Wolbachia* levels.

**Figure 1:**
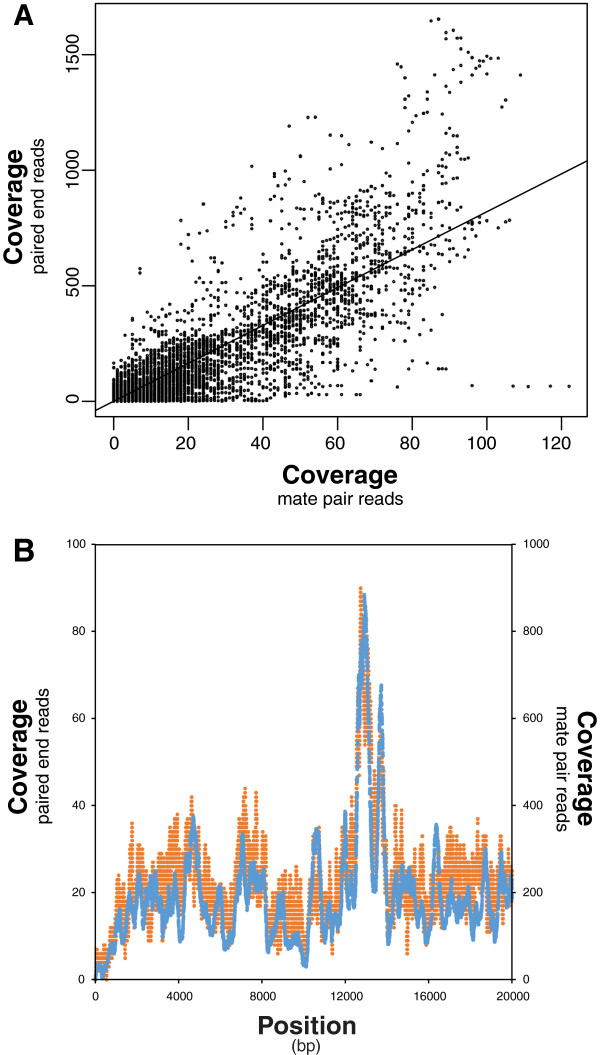
**Correlation between 300-bp paired end reads and 3-kbp mate pair reads. (A)** The coverage measured using the 300-bp paired end reads was correlated with the 3-kbp mate pair reads. While the scatterplot illustrates 100,000 randomly selected points in the dataset, the linear regression was calculated on the entire data set revealing a significant correlation with a p-value of <2e-16, an intercept of 0.61, a slope of 8.2, and an R-squared value of 0.77. **(B)** Overlaying the coverage calculated with the paired end reads (blue) and the mate pair reads (orange) across 20-kbp of the *Brugia* genome shows how the peaks and troughs in both are nearly identical.

**Figure 2:**
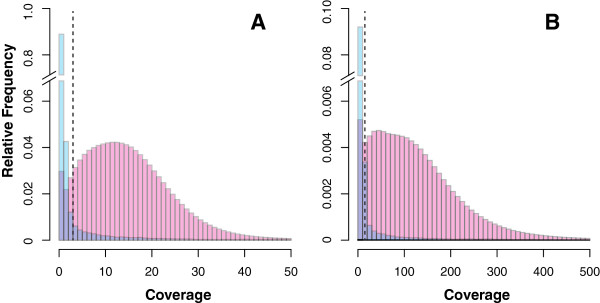
**Coverage distributions.** Histograms illustrate the relative distribution of coverage for mappings to the *w*Bm genome (blue) and the *B. malayi* genome (pink) for BWA-mapped mate pair reads **(**Panel **A)** and paired end reads **(**Panel **B)**. A discontinuous y-axis is used. The *Wolbachia* genome is covered at <2.5× in the mate pair and <15× in the paired end reads. The coverage thresholds used for predicting nuwts are illustrated with dashed lines.

Worms used for sequencing were treated with tetracycline to deplete the *Wolbachia* endosymbionts. A pool of DNA was sequenced with a ratio of *Brugia*:*Wolbachia* DNA >10. Therefore, nuwts should be identified based on a >10-fold difference in coverage relative to coverage of the same sequences in the bacterial genome. More than 49 million 54-bp reads were generated from a 3-kbp mate pair library, and over 138 million 99-bp reads were generated from a 300-bp paired-end library. Given the experimental differences between a 3-kbp mate pair library and a 300-bp paired end library and a significant correlation between the results obtained (Figure 1A; p-value <2e-16), the second sequencing strategy provides independent validation of the first. Previously, in *D. ananassae* we observed differences in the coverage for the paired end libraries and the mate pair libraries, with the mate pair libraries having more smooth coverage and the paired end libraries have a great deal of local variance (data not shown). This was not specifically observed here (Figure 1B) although when compared to the paired end reads (Figure 2A), the coverage distributions are better delineated for the mate pair reads (Figure 2B). Taken together, all subsequent analyses used the paired end data, except where noted.

### BWA analysis

**Figure 3:**
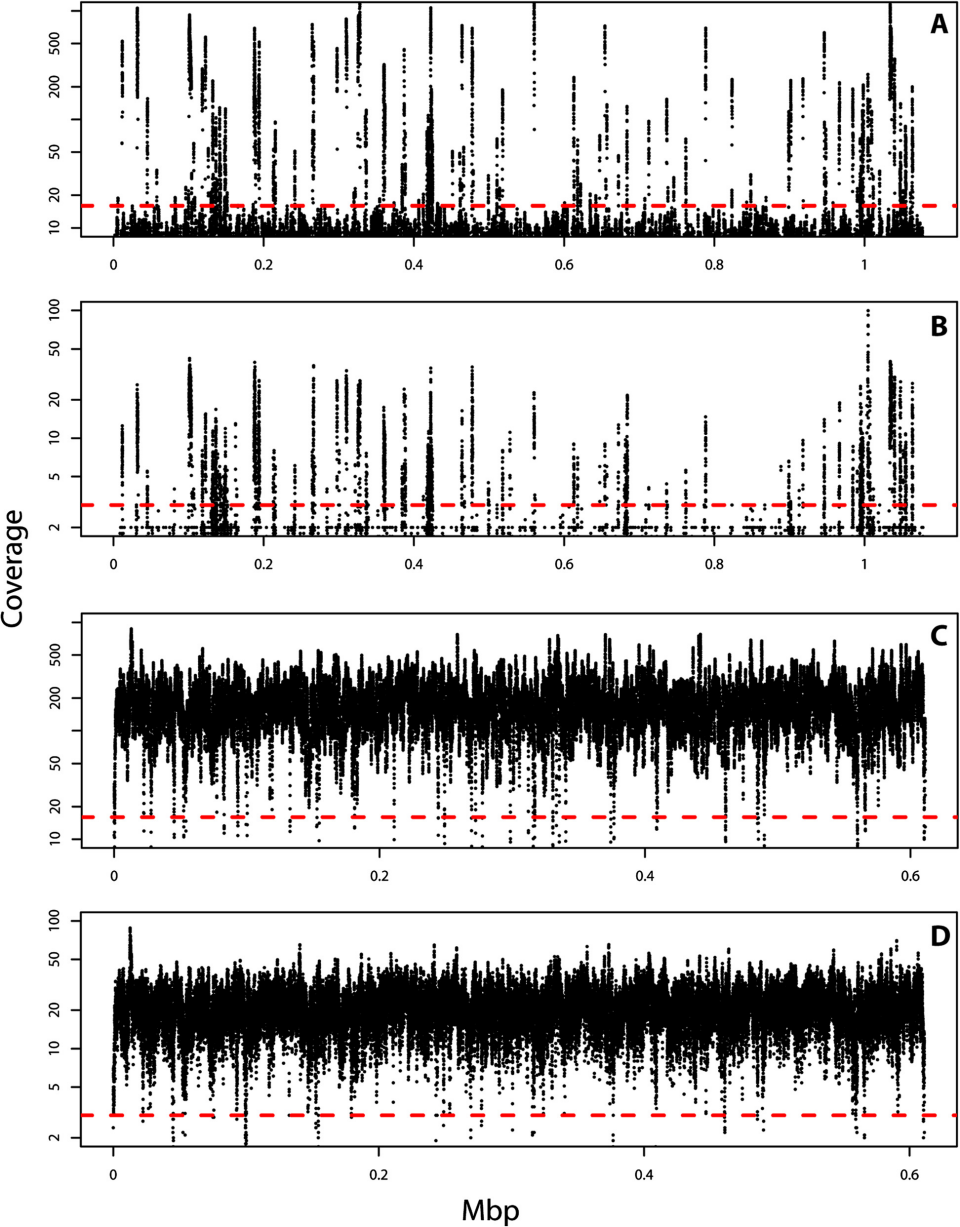
**Coverage across the genome.** The mean coverage along the *w*Bm genome **(**Panels **A** and **B)** or a similarly sized *Brugia* contig **(**Panels **C** and **D)** are plotted using the 300 bp paired end reads **(**Panels **A** and **C)** and the 3 kbp mate pair reads **(**Panels **B** and **D)**. The mean coverage was calculated using 10-bp windows and is presented on a log scale. The dashed red line indicates the cut-off used for detecting nuwts with BWA, which was 16× for the paired end reads and 3× for the mate pair reads. *Wolbachia w*Bm regions having a mean coverage above this cut-off are identified as nuwts located in the genome of *B. malayi*.

The sequencing reads generated were aligned against the *B. malayi* genome [GenBank:AAQA00000000] [17] and the *Wolbachia* strain *w*Bm [GenBank:AE017321] [18] using BWA [19], a short read aligner that is fast and finds only near-identical matches. As such, this version of BWA is best suited for finding evolutionarily recent nuwts with <67 SNPs/kbp [19]. It also natively allows for analysis of mate pairs that can facilitate mapping nuwts to gaps in the *B. malayi* genome when one read in the pair is of *Wolbachia* ancestry and the other read is of nematode ancestry, facilitating some downstream analyses. As expected given the *Wolbachia* depletion strategy, the majority of the *w*Bm genome was shown to have relatively low coverage with nuwts having relatively high coverage, comparable to or greater than the mean coverage of *B. malayi* (Figures 2 &3, Tables 1 &2). More specifically, the mean coverage across *w*Bm was 1.6× and 13.8× with the mate pair and paired end reads, respectively. In contrast, and as expected for *Wolbachia*-depleted worms, the corresponding numbers for *B. malayi* were 16.0× (Table 1) and 130.6× (Table 2). This is consistent with the 10-fold depletion anticipated. Of note, the mean for the *w*Bm mapping is skewed by the numerous high coverage values obtained for nuwts.

**Table 1:** **Coverage of the *Wolbachia w*Bm and *Brugia malayi *genomes for mate pair reads**

	***Wolbachia w*Bm**	***Brugia malayi***
**Mate pair reads mapped (BWA)**	33,874	26,733,712
**Mbp mapped**	1.83	1,443.62
**Mean coverage ± standard deviation**	1.6 ± 7.3	16.0 ± 14.3
**Median coverage**	0	14
**Mode coverage**	0	12
**Maximum coverage**	131	1956

**Table 2:** **Coverage of the *Wolbachia w*Bm and *Brugia malayi *genomes for paired end reads**

	***Wolbachia w*Bm**	***Brugia malayi***
**Paired end reads mapped (BWA)**	158,608	115,356,708
**Mbp mapped**	15.70	11,420.31
**Mean coverage ± standard deviation**	13.8 ± 68.7	130.6 ± 122.3
**Median coverage**	4	109
**Mode coverage**	3	0
**Maximum coverage**	1655	9873

### Detection of nuwts

The critical coverage for distinguishing between *Wolbachia* and nuwts was determined to be 3× for the mate pair reads (Figures 2A &3B, D) and 16× for the paired end reads (Figures 2B &3A, C), as described in the methods. Using these thresholds, the boundaries of nuwts >100-bp were defined relative to the *w*Bm genome using a sliding window approach to smooth coverage variance.

**Figure 4:**
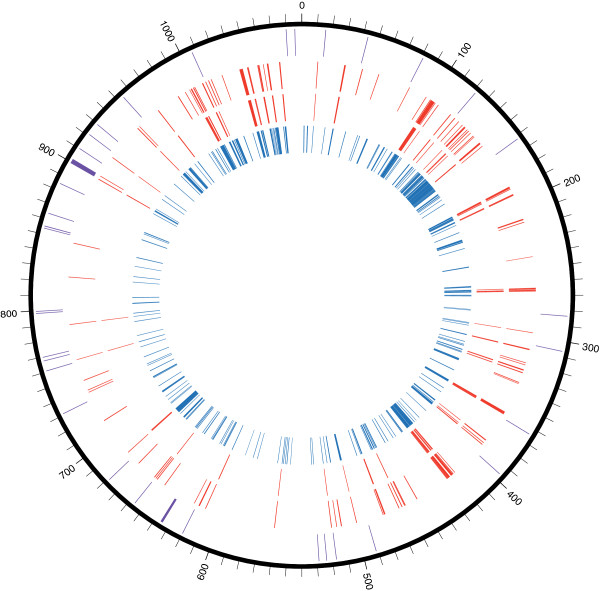
**Position of BWA- and BLASTN-detected nuwts in *Wolbachia w*Bm.** The genome of *Wolbachia* strain *w*Bm is shown in black as a circle. The outermost rim (purple) represents the position of RNA genes. Nuwts are shown that were detected with BWA on paired end data (rim 2), with BWA using mate pair data (rim 3), and with BLASTN using paired end data (rim 4). Some potentially large, nearly contiguous regions >5-kbp can be observed as nuwts on a region spanning ~35-kbp.

Candidate nuwts were covered by 28,060 mate pair reads and 115,812 paired end reads (Table 3). Between 28.6-kbp and 48.8-kbp of the *w*Bm genome was transferred to the *B. malayi* genome as determined using the mate pair and paired end reads, respectively. Given that the *w*Bm genome is 1.08-Mbp, >4.5% of it was present in *B. malayi* as recent nuwts that were detected by BWA mapping of the paired end reads (Figure 4). Furthermore, the genes detected were distributed throughout the *w*Bm genome (Figure 4).

**Table 3:** **Summary of nuwts in *B. malayi***

**Alignment program**	**Sequencing platform**	**Number of reads mapping**^**a**^	**Cumulative length of reads (kbp)**	**Cumulative length on *w*Bm (kbp)**	**Mean coverage threshold**
BWA	GAIIx	28,060	1,515.2	28.6	≥3x
BWA	HiSeq 2000	115,812	11,465.4	48.8	≥16x
BLASTN	HiSeq 2000	628,558	62,227.2	115.4	≥16x

^a^Counts also include reads that may not be located in a nuwt themselves as long as their mate is located in one.

### Experimental verification of nuwt copy number using qPCR

Coverage from the paired end data set was deep enough to estimate nuwt copy number. Considering that average coverage in *B. malayi* was 131× (Table 2), there appear to be multiple copies of specific nuwts in each haploid genome based on coverage (Table 4, Additional file 1). Duplication may suggest that these nuwts are playing a fundamental role in the biology of *B. malayi*, provided that they are functional in *B. malayi*. Copy number variation is linked to phenotypic diversity and evolutionary adaptation [20]. To this end, copy number variation for 10 nuwts was verified using qPCR (Table 4).

**Table 4:** qPCR-based copy number for 10 nuwt fragments, compared to the corresponding coverage-based copy number

**Locus tag**	**Common name**	**Coverage-based copy number for entire gene**	**Coverage for qPCR amplified fragment ± S.D.**	ΔCt ± S.D.*	**qPCR-based copy number**
Wbm0022	alpha/beta fold family hydrolase	6-8	773 ± 63	2.5 ± 0.7 (n = 6)	3-9
Wbm0078	4'-phosphopantetheinyl transferase	5	793 ±42	2.5 ± 0.2 (n = 3)	5-6
Wbm0147	thiol-disulfide isomerase	2-5	287 ± 59	2.4 ± 0.2 (n = 3)	5-6
Wbm0240	HIT family hydrolase	4-6	712 ± 119	2.2 ± 0.4 (n = 3)	3-6
Wbm0241	NADH:ubiquinone oxidoreductase chain A	4	530 ± 22	−0.1 ± 0.2 (n = 3)	1
Wbm0275	glutamine synthetase	2	293 ± 15	−1.7 ± 0.2 (n = 3)	<1
Wbm0429	DNA polymerase III beta clamp subunit	10-12	1288 ± 79	2.5 ± 0.5 (n = 6)	4-8
Wbm0502	50S ribosomal protein L9	3-6	646 ± 188	2.6 ± 0.3 (n = 6)	5-7
Wbm0784	hypothetical protein	3-4	416 ± 21	2.1 ± 0.2 (n = 3)	4-5
Wbm0797	type IV secretion system ATPase VirB4	1-15	162 ± 27	−0.2 ±0.3 (n = 3)	1

* ΔCt relative to the average Ct of six single copy *B. malayi* genes.

**Figure 5:**
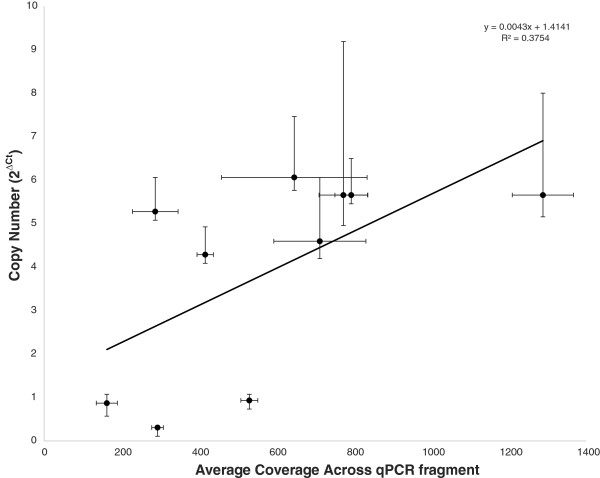
**Correlation between qPCR and coverage.** The average coverage across a fragment amplified by qPCR is compared to the copy number, measured by the ΔCt of the qPCR reaction relative to the average Ct value of six single copy *B. malayi* genes. The error bars for the average coverage are one standard deviation. The error bars for the copy number are derived from one standard deviation of the ΔCt, making them asymmetric since copy number is exponentially related to ΔCt.

For most of the nuwts, the copy number estimate based on read coverage was very close to the qPCR estimate. When the average coverage was compared to qPCR results, a positive correlation was observed (Figure 5, p-value = 0.060) providing a second independent validation of the coverage estimates. The most notable exception was Wbm0241, for which only one copy was seen by qPCR but four copies were estimated by coverage on the genome sequence. One explanation may be that multiple fragmented copies add up to four copies, but only one copy is spanned by the qPCR primers used.

### Size and potential role of transfers

**Figure 6:**
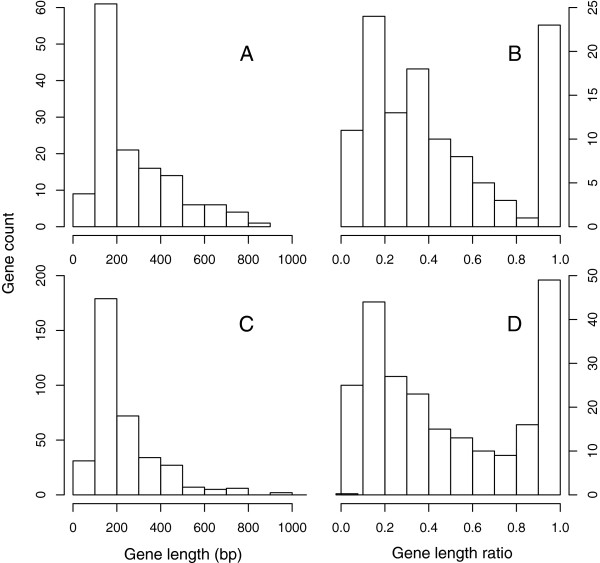
**Size distributions of absolute and relative nuwt length.** A histogram reveals that most nuwts are 100–200 bp. **(**Panel **A)**. A histogram reveals that most nuwts cover either 90-100% of the length of the reference gene, or 10-20% of the reference gene length **(**Panel **B)**. The corresponding distributions for absolute length and proportional length for BLASTN-detected nuwts are also shown **(**Panels **C** and **D**, respectively**)**.

Examining the paired end data set in more detail showed that BWA-detected nuwts in *B. malayi* originated from 116 *w*Bm genes (Additional file 1). Coverage for 21 genes was high across their entire length (Additional file 2), but the remaining genes were only partially covered, suggesting that only part of the respective *w*Bm gene was detected as a nuwt. Frequently, only 100–200 bp of a gene was observed as a nuwt (Figure 6A). Furthermore, a pattern is seen where only a minor fraction of a gene is present (e.g. 20% of the gene) or the entire gene is present (Figure 6B). This suggests that some transfers include full-length genes. However, a substantial number of gene fragments are transferred or many of the full-length genes transferred have decayed yielding gene fragments.

No significant difference (Pearson’s Chi-squared test, Yate’s continuity correction, p > 0.53) was observed between the frequency of COG functional categories in all nuwts or full-length nuwts when compared to those for *w*Bm. However, genes not classified in any COG category were significantly under-represented in nuwts (26/104) relative to the *Wolbachia w*Bm genome (297/805) (Pearson’s Chi-squared test, Yate’s continuity correction, p = 0.02).

### Nuwt location in the *B. malayi* genome

To examine the insertion sites of nuwts in the nematode genome, we identified read pairs spanning the junction between the nuwt and the portion of the *B. malayi* genome of nematode ancestry. Such pairs representing junctions have one read mapped to the *w*Bm genome and one to the *Brugia* genome. As expected, significantly more junctions were present in intergenic regions and significantly fewer junctions in coding sequences and introns/UTRs (Table 5; Pearson’s Chi-squared test, Yate’s continuity correction, p < 0.01), although junctions near genes could be identified.

**Table 5:** **Nuwt location in the *B. malayi *genome**

**Genomic feature**	**Reads mapping**	**Even distribution**	**%**	**p-value**
Coding sequences	599*	1522	39.4%	< 2.2e-16
Introns / Untranslated regions	549*	2343	23.4%	< 2.2e-16
Intergenic regions	7803*	5356	145.7%	< 2.2e-16
Contig ends	1517*	1247	121.7%	4.0e-08

*Denotes significant difference (Pearson's Chi-squared test, Yate's continuity correction, p < 0.01.

The number of junctions near the ends of contigs differed significantly from a uniform distribution (Table 5; Pearson’s Chi-squared test, Yate’s continuity correction, p < 0.01). During assembly of the reference genome, sequence reads were removed that had >98% identity to *w*Bm >90% of the length of the read. Since the sequences examined here were mapped with BWA, they lack polymorphisms and they likely represent the sequences removed with such criteria as was used for removing *w*Bm sequences prior to assembly. Given an overabundance of nuwts near contigs ends, it is likely that some of the gaps in the *Brugia* genome resulted from removal of sequences with near identity to *w*Bm, and that these gaps may be filled with nuwt sequence.

### Fixed polymorphisms

BWA alignment of HiSeq reads against the *w*Bm genome showed that most of the 125 nuwts contained at least one single nucleotide polymorphism (SNP). A subset of these SNPs had only one polymorphism and as such, are likely fixed. This suggests that nuwts have been accumulating over recent time in *B. malayi.*

### BLASTN analysis

We also searched for nuwts of different evolutionary ages using BLASTN [21]. BLASTN was selected because it is still relatively fast, but it detects matches with higher levels of polymorphisms when compared to BWA. This makes it more sensitive and suitable for detecting older nuwts. However, BLASTN does not perform well on short reads and therefore only the 99-bp paired end reads were analyzed in this manner.

BLASTN was used to detect hits with >80% similarity. While a lower stringency is possible in a BLASTN analysis, we have found that lowering the stringency in this case yields matches to genes annotated as arising from the mitochondria, or numts. This is a peculiar result given the ancient ancestry of mitochondria and is under further, separate investigation. Regardless, this observation also precluded the use of a TBLASTX-based analysis.

With a coverage cut-off of 16×, 115.4-kbp (or 10.6%) of the *w*Bm genome is identified using BLASTN, including fragments of 227 *w*Bm genes (Additional file 3). Thirty-two of these genes had their entire length covered by nuwts, making them excellent candidates for downstream functional analyses (Additional file 2, Figure 6C, D). Like with the BWA-based analysis, no COG category was found significantly different in genes found in nuwts, compared to the complete set of *w*Bm genes (Pearson’s Chi-squared test, Yate’s continuity correction, p > 0.29).

The BLASTN analysis greatly increased the fraction of the genome of *w*Bm implicated in LGT events. More specifically, when BWA-detected and BLASTN-detected LGTs are compared to each other, there is an additional 65.9-kbp of the *w*Bm genome present as nuwts. Nuwts in this additional genome portion included new fragments of 162 genes. This demonstrates that nuwts have a continuous distribution of nucleotide divergence suggesting that the transfer of *Wolbachia* DNA to the genome has occurred over a long time span. It is possible that transfers have occurred since the origin of the symbiosis.

### Transcriptional activity of nuwts

Transcription of nuwts containing full-length *w*Bm genes was examined using publicly available RNA-Seq data [22]. The *B. malayi* transcriptome was sequenced in 13 samples corresponding to 7 different life stages [22]. When sequences from each of these samples were aligned against the *w*Bm genome it was found that up to 0.23% of the reads were mapped. This finding was unexpected since poly-A selection took place before sequencing [22], and that step should exclude bacterial transcripts. Such RNA-Seq reads may arise from nuwt transcription. Supporting this hypothesis, a comparison of the mean coverage of the nuwt regions to that of the non-nuwt regions showed a small but highly significant difference between them (Wilcoxon rank sum test with continuity correction, p < 2.2×10^-16^ for all 13 samples). The ratio of the average coverage for nuwts compared to non-nuwt regions of the *w*Bm genome varied among the thirteen samples from 1.8 to 7.0. This statistically significant difference means that transcripts with similarity to *Wolbachia* are more likely to arise from nuwts than from the bacterial genome.

To further validate whether transcripts are arising from the nuwts, the RNA-Seq data were examined for nuwt-specific polymorphisms (Additional file 4). The 21 BWA-detected, full-length nuwts were particularly interesting since they were more likely to have retained some function. Fourteen of them had strong evidence for transcription in at least one life stage, when examining nuwt-specific SNPs (Additional file 5). More specifically, for some *Wolbachia w*Bm genes all reads found contained only the nuwt-specific SNP, which means that transcription comes only from the nuwt copy of that gene (Additional file 4).

**Figure 7:**
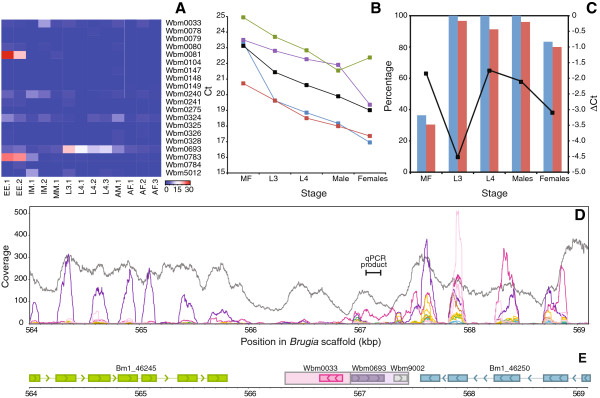
**Transcriptional activity of nuwts. (A)** Transcription levels of full-length protein-coding nuwts are illustrated with a heatmap of RPKMs on a blue-white-red gradient using publicly available data [22]. Blue represents no expression and red represents the most expression with six stages examined: EE, eggs and embryos; IM, immature microfilariae; MM, mature microfilariae; L3, 3rd stage larvae; L4, 4th stage larvae; AM, adult males; AF, adult females. Wbm0693 (hypothetical protein) is highly transcribed in L3 stage infective larvae, and Wbm0081 and Wbm0783 are highly transcribed in eggs/embryos. **(B)** Four genes were shown to be constitutively expressed and were subsequently used in experiments to validate the results with Wbm0693: green, Bm1_03960, ribosomal protein L16; purple, Bm1_03910, 40S ribosomal protein S27; blue, actin; red, tpH-1; black, average. **(C)** Relative abundance of the nuwt originating from Wbm0693 is illustrated relative to the average of the four control genes from panel **B** (black line). The amplified region of the nuwt contains two SNPs that differentiate the nuwt copy of the gene from the *Wolbachia* copy allowing the percentage of the clones containing high-quality, nuwt-specific SNPs (blue bars) and the percentage of positions containing high-quality, nuwt-specific SNPs (red bars) to be calculated. The vast majority of the transcripts in the L3, L4, male, and female stages originate from the nuwt copy. **(D)** The paired end reads generated in this study (gray) and the publicly available RNASeq data (EE, green; IM, light blue, MM, red; L3, magenta; L4, orange; AM, purple; AF, pink) were mapped to the region in the *B. malayi* genome containing Wbm0693. The region amplified in the qRT-PCR experiments presented in Panels **B** and **C** is also illustrated. **(E)** Genomic features are illustrated for the same region as in Panel **D**.

Subsequently, transcription levels were calculated as RPKM values [23] for each of these 21 genes. Stage-specific expression is seen with Wbm0693 transcribed in L3 larvae and Wbm0081 and Wbm0783 transcribed in eggs/embryos (Figure 7A). All three genes are annotated as hypothetical proteins.

The nuwt originating from the Wbm0693 gene was further examined using quantitative, reverse transcription PCR (qRT-PCR) on RNA from microfilaria, L3, L4, adult males, and adult females. The qRT-PCR product for Wbm0693 was 16-64× more abundant across all five stages than two hypothetical proteins (Wbm0149 and Wbm0783) that are present as nuwts but only expected to be transcribed by the bacteria based on SNPs in the transcriptomics sequence. Wbm0693 is 1-32× less abundant than *groEL*, which was not identified as a nuwt, but is an abundant transcript in most intracellular bacteria and was amongst the most abundant *Wolbachia* proteins identified in a proteomic analysis of *B. malayi*[24]. Wbm0693 is 2-16× less abundant than the average transcript level for 4 constitutively expressed genes (Figure 7B, C), but is of similar abundance to two of these constitutively expressed genes of nematode ancestry (Bm1_03910 and Bm1_03960).

The analysis of transcription is complicated since the qRT-PCR product could originate from RNA from either the bacteria or the nuwt. Not only do *Wolbachia* numbers change throughout the nematode life cycle, but transcripts from both origins will have differential transcription through the different life stages. Therefore, the Wbm0693 amplicons were cloned and sequenced, and quantification of the nuwt-specific SNPs was used to identify the relative contributions of the nuwt and bacterial transcripts. While the transcript abundance is lowest in the L3 as measured by the ∆Ct, 100% of the amplicons arise from the nuwt (Figure 7C). In contrast, transcription is high in microfilaria, but most of the amplicons arise from the bacteria (Figure 7C). Surprisingly, the transcription was high in the L4, males, and females in the qRT-PCR and was predominated by the nuwt-specific alleles. This is contrary to the transcriptomics data, which had higher transcription in the L3s (Figure 7A). This could reflect biological or technical differences in the RNA obtained for the RNAseq and the qRT-PCR experiments.

The region of the genome that includes Wbm0693 was properly assembled in the original genome sequence, enabling further examination of the transcriptional profile in this region using the RNA-Seq data. The region between Bm1_46245 (hypothetical protein) and Bm1_46250 (apacd-prov protein) contains two adjacent nuwts that arise from different portions of the *Wolbachia* genome. While the flanking genes of nematode ancestry (Bm1_46245 and Bm1_46250) have clear transcriptional profiles indicating the intron/exon boundaries and stage-specific transcription (Figure 7D, E), the nuwt containing Wbm0033 (Figure 7E, pink) is transcriptionally silent. Wbm0033 is a small hypothetical protein with homology to DnaJ heat shock proteins. The other nuwt (Figure 7E, lavender) is the one transcribed in the L3 transcriptomic experiment and contains Wbm0693 and Wbm9002, which are predicted to encode a hypothetical protein and the 5S rRNA, respectively. Different regions of this latter nuwt show different transcriptional profiles.

On the right side of this nuwt is a region encoding the bacterial 5S rRNA, and it is detected in several stages. Since rRNA is quite abundant, this level could reflect endosymbiont rRNA that co-purified with the polyadenylated RNA. The nuwt 5S rRNA has a 14-bp insertion relative to the bacterial-encoded 5S rRNA. This insertion prevents mapping of sequence reads. In all but the L3, transcription levels drop at this 14-bp insertion, supporting that these reads arise from the bacteria-encoded 5S rRNA in all stages except L3. However, the reads from L3 contain this 14-bp sequence, supporting that the transcription in the L3 is from the nuwt.

On the left side of this nuwt is a region encoding Wbm0693. The 5′-portion of Wbm0693 is transcribed in numerous stages, but the 3′-portion is transcribed only in L3. The transcription in L3 is evident across the entire nuwt and into the adjacent gene, Bm1_46250. Since the directionality of the transcripts was not assessed in the RNA-Seq experiments, it is not possible to determine if this transcription results from a promoter activating transcription of the nuwt or if there is alternative splicing of Bm1_46250 that leads to transcription of this region. The latter would result in anti-sense transcription of Wbm0693 and a chimeric mRNA. The former could result in an mRNA that codes for Wbm0693 or alternatively could result in transcriptional interference [25,26]. The resulting protein would be full length but would have a 7-aa insertion.

## Discussion

Lateral gene transfer in eukaryotes is a rare phenomenon, likely because the eukaryotic germline is segregated from the other tissues. This makes the numerous interdomain LGT events found between *Wolbachia* and its eukaryotic hosts intriguing [4,7-15]. An advantage *Wolbachia* has in donating DNA is that it is found in the reproductive tissues and embryos of its hosts. This means that it is ideally positioned for creating heritable LGT in its eukaryotic hosts. The sizes of known nuwts range from a few hundred bp to the entire *Wolbachia* genome [4,7-15]. In this study, we undertook deep sequencing of *B. malayi* nematode worms and compiled a more complete list of nuwts in *B. malayi*. Such detailed cataloguing of the *B. malayi* nuwts enabled the study of their potential functionality as well as their frequency.

No particular COG class could be found that was overrepresented in the nuwts. However, genes without a function were under-represented. This former result may suggest that there is no preference for the genes that get transferred and that the entire *Wolbachia* chromosome is potentially transferrable. The latter result may reflect that LGT in *Wolbachia-*nematode systems is RNA-mediated. Previously, proteomics studies have established that ≥99% of the genes with a function are expressed in the closely related bacteria, *Ehrlichia chaffeensis* and *Anaplasma phagocytophilum,* while only ~80% of hypothetical proteins are expressed [27]. If the same is true in *Wolbachia,* this bias in genes with and without a function may reflect that LGT occurs through transcripts, and is RNA-mediated, possibly through retrotransposition. This is also consistent with the size of the transfers observed that are similar to the size and composition of bacterial transcripts from operons. This is in contrast to *Wolbachia*-insect LGT, where large chromosomal fragments are frequently found that must be DNA-mediated. Recently, evidence has been presented to demonstrate LGT from bacteria to the human somatic genome, possibly through an RNA-intermediate [28]. This observation in humans correlates well with what is known about the recognition of RNA molecules by the human immune system [28]. If such preference for an RNA-intermediate in nematodes and a DNA-intermediate in insects exists, it would be interesting if it relates to fundamental differences in the nematode and insect immune systems.

### Potential functionality of nuwts

If nuwts are simply decaying after their integration into the eukaryotic genome, then they will not be functionally significant. We established transcription for several of the nuwts examined, however transcription does not necessarily imply function [5] and it appears that low-level transcription is common among nuwts [4,11-13]. Using publicly available RNA-Seq data [22], it was found that at least three of the full-length nuwts are transcribed in a life stage-specific manner and at levels that could be biologically meaningful. Life stage-specific transcription, as opposed to constitutive transcription, can be an additional indicator of potential functionality [5].

Analyses like gene silencing are needed to conclusively establish if the nuwts are functional. There are several examples of functional nuwts. In the first case, genes of ancestry that may include *Wolbachia* are found in the genome of the pea aphid *Acyrthosiphon pisum*, which is a *Wolbachia*-free insect [15]. Some of these genes are related to murein metabolism, have acquired spliceosomal introns, and have tissue-specific transcription.

The second case of a functional putative nuwt is that of salivary gland specific (SGS) genes of the mosquitoes *Aedes aegypti* and *Anopheles gambiae*, which are associated with *Plasmodium* invasion of the salivary glands of female mosquitoes [8,10,29]. SGS genes do not have similarity with any other eukaryotic genes in the database, and the only related database sequences with homology are from *Wolbachia* endosymbionts [8]. Nuwts in these two systems feature traits that are characteristic of functional nuwts [5,30]: (a) longevity after the LGT event, (b) integration into host genome (for *A. pisum* nuwts) and (c) an associated phenotype (for *Ae. aegypti* nuwts).

### Multiple-copy nuwts

Copy number variation has been suggested to be of great evolutionary importance. More specifically, gene copy number facilitates evolution of new variants of a gene and can also affect transcription levels [31]. In this respect, it is interesting that a considerable number of *B. malayi* nuwts appear to have multiple copies. These copies could result from: (a) repeated transfers of the same genome fragment, (b) duplication of nuwts following the initial LGT event, or (c) some combination of the two. Unfortunately, we were not able to reliably deduce the sequence of each copy, which would provide better insight on the underlying mechanism of this copy number variation. It is worth mentioning, however, that in another case of LGT, an increase in copy number of the transferred genes was detected [32]. These extra copies were interpreted as being part of the adaptation process of the host organism to the newly acquired genes. Hence, studying the mechanism by which the multiple-copy *B. malayi* nuwts arose would further elucidate their evolutionary significance and may become possible when new sequencing technologies become available.

### Potential drug targets

Treatment of lymphatic filariasis has recently included drugs targeting *Wolbachia* rather than the nematode itself [3]. However, there is still the need to develop antifilarial drugs that will offer alternative treatment routes. Certain nuwts found in the framework of this study contained full-length *w*Bm genes and, thus, could represent potentially functional transfers. More specifically, seven of the genes are interesting because of their putative functions. These genes include Wbm0078 (phosphopantetheinyl transferase), Wbm0079 (prolipoprotein diacylglyceryl transferase), Wbm0080 (SsrA-binding protein), Wbm0147 (thiol-disulfide isomerase), Wbm0148 (thymidilate synthase), Wbm0240 (HIT family hydrolase), and Wbm0275 (glutamine synthetase). Intriguingly, the lipoprotein biosynthesis pathway, in which Wbm0079 is involved, has been previously shown to be a valid drug target [33]. In addition, genes Wbm0081, Wbm0693 and Wbm0783 are of special interest, because transcripts for all three have been detected with differential expression in eggs and larvae (Figure 7). Further functional studies using gene silencing are underway to determine if nuwts can be validated as potential drug targets and to further unravel the complexity of *Wolbachia-*filarial symbiosis.

## Conclusions

Our results suggest that >4.5% of the *Wolbachia w*Bm genome has been transferred to the genome of its nematode host, *B. malayi*. A considerable number of *Wolbachia* genome fragments are present in multiple copies in *B. malayi*. At least 21 full-length genes have been laterally transferred. Analysis of existing transcriptomics data suggests that three of the nuwts are highly transcribed in specific life stages. Taken together, these data suggest that some of the nuwts identified could be functional and may be exploited as potential targets for drug discovery.

## Methods

### Generation of *Wolbachia*-depleted *Brugia malayi*

*B. malayi* worms were obtained as described previously [34]. Briefly, adult *B. malayi* were maintained in the peritoneal cavities of jirds (*Meriones unguiculatus*). Worms were depleted of *Wolbachia in vivo* by treating infected jirds with 2.5 mg/mL tetracycline hydrochloride (Sigma) in drinking water for a period of six weeks. Adult *B. malayi* were recovered by dissection two weeks following the end of treatment (eight weeks post-treatment) and maintained until processing in RPMI-1640 supplemented with 2 mM L-glutamine, 25 mM HEPES, 100 U/mL penicillin, 100 μg/mL streptomycin and 2.5 μg/mL amphotericin B. Worms were then separated by sex, rinsed in PBS, and added individually to RNAlater solution (Ambion, Austin, TX, USA) for storage at 4 °C prior to DNA preparation.

### Preparation of DNA and assessment of *Wolbachia*-depletion

Genomic DNA was isolated from individual tetracycline-treated *B. malayi* adult worms using the QIAamp DNA Microkit (Qiagen, Valencia, CA, USA) with overnight lysis and elution in 50 μL of buffer AE. DNA quality was assessed by agarose gel electrophoresis, and quantification was conducted using the Quant-iT PicoGreen dsDNA kit (Invitrogen, Grand Island, NY, USA). Although the tetracycline treatment regimen can yield a 99% reduction in *Wolbachia* over the population [34], the degree of reduction varies between individual worms. Therefore, quantitative PCR targeting the single-copy genes *wsp* of *Wolbachia* and *gst* of *B. malayi*[35] was conducted to determine those individual worms with the lowest *wsp*:*gst* ratios. DNA from individuals with *wsp*:*gst* ratios less than 1:10 were pooled according to sex and used for sequencing.

### Sequencing of *Wolbachia*-depleted genomic DNA from *B. malayi*

Both a 300-bp paired-end and an ~3-kbp mate-pair library were constructed for sequencing on the Illumina platform. The 300-bp paired-end library was constructed using the NEBNext® DNA Sample Prep Master Mix Set 1 (New England Biolabs, Ipswich, MA), while the mate-pair library followed the Illumina Mate Pair Library v2 Sample Preparation Guide protocol. In both cases, DNA was fragmented with the Covaris E210 and libraries were prepared using a modified version of the manufacturer’s protocol. The DNA was purified between enzymatic reactions and the size selection of the library was performed with AMPure XT beads (Beckman Coulter Genomics, Danvers, MA). The PCR amplification step was performed with primers containing 6-bp index sequences. Since short reads are required for mate pair libraries, the mate pair library was sequenced on an Illumina Genome Analyzer IIx while the paired end library was sequenced on an Illumina HiSeq2000. Base calling and quality scoring was performed using Illumina software followed by in-house quality assessment and control pipelines to truncate and eliminate poor-quality reads. All of the sequencing data is available in SRA051817.

### BWA analysis

Both the mate pair (24,864,076 × 2) and paired end (69,289,764 × 2) reads were used for finding evolutionarily recent LGTs from *Wolbachia w*Bm to *B. malayi*. Mate pair reads were first reverse-complemented using the FASTX-toolkit [36]. Then both mate pair and paired end datasets were mapped on *Wolbachia w*Bm [GenBank:NC_006833] as well as on *Brugia malayi* [GenBank:AAQA00000000] genomes using BWA 0.5.9-r16 [19] with the default parameters. The resulting SAM files were then processed using SAMtools [37] for discarding non-mapping reads and sorting. MarkDuplicates from Picard-tools [38] was used for removing read duplicates. The output BAM files were used to generate a file of coverage per base in VCF format [37] with *Wolbachia w*Bm or *B. malayi* as the reference genome. Subsequently, we extracted read coverage at each genomic position and calculated, using R [39], the mean coverage of our reads on the genomes of *w*Bm or *B. malayi* (Table 1, Table 2).

To select an appropriate coverage cutoff for distinguishing between nuwts and *Wolbachia* sequences, coverage at each *w*Bm position was extracted using the sorted BAM file as input to BEDtools [40]. Subsequent examination of the coverage distribution showed that there were a large number of positions with coverage below 3× for mate pair reads (Figure 2A) and below 16× for paired end reads (Figure 2B). Such low coverage positions originated from the *Wolbachia* endosymbiont. In contrast, positions that were covered highly did so because they were actually located in the chromosomes of *B. malayi* where coverage is high. Hence, 3× and 16× were selected as coverage cut-offs for detecting nuwts, for mate pair and paired end reads, respectively.

In order to determine the *w*Bm regions that were transferred to *B. malayi*, a sliding window approach was followed, using 10-bp windows with a 5-bp step. Only high coverage windows were extracted using the 3× and 16× cutoffs (as explained above) as a measure of nuwts. Based on paired end data, some nuwts were found to be present in multiple copies in *B. malayi*. The copy number, per haploid *B. malayi* genome, was estimated by dividing the mean coverage of the respective nuwt by the mean coverage of *B. malayi* (i.e. 131 for paired end reads). Lastly, recent nuwts were plotted on the circular chromosome of *w*Bm using Circos [41].

### Establishing criteria for defining nuwts

The statistical tests that can be used to distinguish nuwts from the bacterial genome are limited because the data is neither unimodal nor normal, as assessed by the differences between the mean, mode, and median for the data (Tables 1 and 2). Theoretically, statistics used to predict the modes (peaks) and antimodes (troughs) in a histogram of coverage (Figure 2) may prove suitable, if all nuwts were single copy. However, the coverage measurements suggest many multi-copy nuwts and as such a multi-modal method is required.

Mode-based analyses quickly become cumbersome and difficult to derive for multi-modal distributions. A multi-modal distribution would be expected if numerous nuwts have different copy numbers. The major mode will reflect the coverage across the bacterial genome while there will be numerous minor modes. Theoretically, one of these minor modes will reflect nuwts at a single copy in the *B. malayi* genome and should be similar to the distribution of coverage found for single copy *B. malayi* genes. In addition, other minor modes occur that represent the various copy numbers represented by the nuwts.

Assuming one could derive the statistics for the number of modes observed, the data has several other problems as it relates to an analysis of modes. The major mode is of a significantly higher magnitude than the minor modes while the minor modes have a higher standard deviation, assuming they have the same standard deviation as the mappings to the *Brugia* genome. This results in a histogram where the minor modes appear as a shoulder to the major mode and to one another.

Given these observations about the data, an appropriate statistical test to dissect the two coverage distributions could not be identified. Therefore, the antimode was established empirically by visually inspecting the coverage and the histogram of the coverage across both genomes (Figures 2 and 3). With this examination one can arrive at a suitable value for the antimode between the major mode in the coverage distribution using the *w*Bm genome (Figure 2, blue) and an estimate of the nearest minor mode using the position and breadth of the coverage distribution across the *B. malayi* genome (Figure 2, pink). The ideal position would maximize the number of observations made while minimizing the number of false observations. Since the coverage on the *w*Bm genome drops precipitously at the same point that the coverage on the *Brugia* genome is slowly increasing, the ideal cut-off was determined to be immediately to the right of the precipitous decline. In addition, this cutoff is approximately one standard deviation below the mean for the *Brugia* coverage. Of note, the use of standard deviation is not ideal since the *Brugia* coverage is also not normal or unimodal (Figure 2) likely owing to the fact that the reference genome is not complete. For instance, regions of no coverage are observed in the histogram that likely reflects gaps in the contigs.

### Over- or under-representation of COG classes in nuwts

To examine if certain functions were preferentially involved in LGT to the host, all *w*Bm genes were assigned COG (Cluster of Orthologous Group) classes [42]. The frequency of each COG class was counted, as well as the frequency of genes having no COG classes. The same frequencies were counted only for the subset of genes appearing either in BWA- or BLASTN-detected full-length nuwts. Significant differences in COG frequencies were identified using Pearson's Chi-squared test with Yate's continuity correction and a p-value threshold of p < 0.01.

### Nuwt location in the *B. malayi* genome

Detection of the nuwt insertion sites was based on BWA mappings because BWA keeps track of mate pairs. Only paired end reads were used because they were derived from a 300-bp library and, hence, defined more precisely the junction boundaries.

As a first step, reads not mapped in the *Wolbachia w*Bm genome were extracted if their mates were mapped. Next, these reads were mapped against the *B. malayi* scaffolds [GenBank: DS236884-DS264093] using BWA with the default parameters. Finally, using the GFF file for *B. malayi* scaffolds, we categorized mapped reads based on their location in the genome. More specifically, reads were placed in one of the following categories; (a) coding sequences, (b) introns/UTRs, (c) intergenic space, and (d) contig ends. Only one category was assigned to each read.

A statistical analysis was undertaken to compare the distribution in each category to an even distribution across the genome. We counted the number of base pairs found in each of the four categories for the entire genome. We subsequently dealt with each category separately, comparing the actual number of junctions found in that category to the expected number of junctions if it were a purely random process, using Pearson's Chi-squared test with Yate's continuity correction with a p-value threshold of p < 0.01.

### Experimental verification of nuwt copy number

A nuwt copy number estimate was determined by dividing coverage of each nuwt by 131, or the single copy coverage of the haploid *B. malayi* genome. To check the accuracy of the coverage-based estimates, 10 nuwts were selected and their copy numbers were experimentally determined using qPCR. An additional 12 genes including 6 for *w*Bm and another 6 genes for *B. malayi* were selected as single-copy control genes (Additional file 6). Primers for amplifying a 100–150 bp product were designed using Primer3 [43] and synthesized by Sigma-Aldrich (St. Louis, MO, USA).

*B. malayi* genomic DNA (70 ng) was used as template in a qPCR reaction containing 2X QuantiTect SYBR Green (Qiagen), RNase-free water, and coverage primers using the standard Qiagen SYBR Green PCR protocol (Qiagen, Germantown, MD, USA). The assays were conducted using an ABI 7900 instrument (Applied Biosystems, Foster City, CA, USA). The reactions were denatured at 95 °C for 15 min followed by amplification with 45 cycles of 94 °C for 15 s, 55 °C for 30 s and 72 °C for 30 s. Reactions were followed by a melt curve analysis that starts at 55 °C, with a dissociation step at 95 °C for 1 min plus 0.5 °C/cycle for 80 cycles. Copy number was determined by relating the average abundance of the 12 single-copy amplicons to the average abundance of the 10 nuwts as 2^( ΔCt (single copy - nuwt)). Three technical replicates were performed.

### Detecting fixed polymorphisms

There were 106 of the 125 BWA-detected nuwts that had single nucleotide polymorphisms (SNPs), compared to the reference *w*Bm genome. A subset of these nuwts had fixed SNPs meaning the difference compared to the reference was the same in all reads. Fixed polymorphisms were examined with SAMtools mpileup [37] when supported by at least 20 reads. Positions containing gaps were excluded. From the remaining nuwt positions we extracted those in which the number of reads differing from the reference was greater than 80% of the total number of reads. The remaining 20% were assumed to arise from the bacterial sequences.

### BLASTN analysis

Only the Illumina paired end read set (138,579,528 99-bp reads in total) was mapped on *Wolbachia w*Bm using BLASTN [21] with an e-value cutoff of 10^-5^. All reads were treated as single end reads. Hits shorter than 45 bp were filtered out and only the best e-value hit of each query was kept. Using an in-house Perl script, the BLAST output was converted to a SAM file that was sorted with SAMtools [37]. Coverage was then calculated using the VCF output of SAMtools mpileup [37].

A coverage cutoff of 16× for detecting older nuwts was selected using a method identical to the one used in the BWA analysis and plotted on the circular chromosome of *w*Bm using Circos.

### Transcription of nuwts

Transcription of nuwts was examined using RNA-Seq data for *B. malayi* found at Array Express (http://www.ebi.ac.uk/arrayexpress/) under accession number E-MTAB-811 [22]. The 13 different fastq files available correspond to 7 different life cycle stages of the worm. All files were searched against the *Wolbachia* sp. *w*Bm genome using BWA with the default parameters. The resulting SAM file was processed in the same manner as the BWA analysis of DNA sequences in order to produce a VCF file using SAMtools mpileup [37]. Using custom Perl scripts, *w*Bm genomic positions were tagged as belonging either to nuwts or non-nuwts and the coverage was also extracted. Significance was assessed using the R statistical package (v. 2.14.1) for transcript coverage of nuwts relative to non-nuwts.

Transcription levels for full-length nuwts were calculated as RPKM values [23]. Briefly, all reads found inside each such nuwt were counted and subsequently normalized by the gene length and also by the number of reads mapping against the scaffolds of the *B. malayi* genome. Subsequently, heatmaps were drawn using the R package (v 2.14.1), to illustrate highly transcribed genes.

### Quantitative RT-PCR

Quantitative RT-PCR (qRT-PCR) was used to validate the publicly available RNA-Seq data using RNA provided by the NIAID FR3 Resource Center. Reverse transcription was carried out using the QuantiTect Reverse Transcription Kit (Qiagen, Valencia, CA, USA) in accordance with the manufacturer’s instructions. Briefly, ~100 ng of total RNA was incubated at 42 °C for 2 min in genomic DNA wipeout buffer and RNase free water. The cDNA was synthesized from the RNA using Quantiscript reverse transcriptase, Quantiscript RT buffer and a primer mix consisting of long random primers and oligo-dT. The reaction was incubated at 42 °C for 15 min and then at 95 °C for 3 min to inactivate the Quantiscript RT. Real-time detection was carried out on 9.1 ng of the resulting cDNA in a reaction containing QuantiTect SYBR green mix (Qiagen, Valencia, CA, USA), RNase free water, and gene-specific primers (Additional file 7) on a ABI7900HT machine (Applied Biosystems, Beverly, MA, USA). The reactions were denatured at 95 °C for 15 min followed by amplification with 45 cycles of 94 °C for 15 s, 55 °C for 30 s and 72 °C for 30 s. The qRT-PCR data was analyzed using a comparative cycle threshold (∆Ct) method. The Ct value of the *Wolbachia* and nuwt reactions were compared to that of four constitutively expressed *B. malayi* controls. To determine the relative contributions of the nuwt RNA and the bacterial RNA, reactions without SYBRGreen were set-up in parallel that were cloned using the TOPO-TA cloning kit for sequencing (Invitrogen, Carlsbad, CA, USA) and transformed into TOP10 cells (Invitrogen, USA). Colonies were picked, grown in LB with kanamycin, and plasmids sequenced at the University of Maryland School of Medicine Genome Resource Center (Baltimore, MD, USA) with the M13F and M13R primers.

### Availability of supporting data

The sequencing reads supporting the results of this article are available in the short read archive, SRA051817 at http://www.ncbi.nlm.nih.gov/sra/?term=SRA051817.

## Competing interests

The authors declared that they have no competing interests.

## Authors’ contributions

PI performed the computational analyses, qPCR experiments to analyze copy number, and also drafted the manuscript. KLJ created *Wolbachia*-depleted nematodes and isolated their genomic DNA. DRR performed computational analyses and drafted portions of the manuscript. NK performed qPCR experiments to analyze copy number, qRT-PCR experiments, cloning, and drafted portions of the manuscript. JRW performed the statistical analyses. KTO performed qPCR experiments to analyze copy number. SO carried out sequencing of genomic DNA from *Wolbachia*-depleted worms. LJT assisted in designing the sequencing experiments and oversaw sequencing. JMF and MJT designed the experiments. JCDH conceived of the study, participated in its design and coordination, and drafted the manuscript. All authors read and approved the final manuscript.

## Supplementary Material

Additional file 1 List of BWA-detected nuwts. Click here for file

Additional file 2 **Comparison of *Wolbachia w*Bm genes completely covered by BWA- and BLASTN-detected nuwts.** Click here for file

Additional file 3 List of BLASTN-detected nuwts. Click here for file

Additional file 4 **Nuwt transcription based on nuwt-specific SNPs.** The fields in this file correspond to the following: (1) *Wolbachia w*Bm accession number, (2) nuwt coordinate, (3) *w*Bm gene overlapping with the SNP (“ig” = intergenic region), (4) SNP position in the reference genome, (5) base in the reference genome, (6) sequencing coverage of the *w*Bm genome, (7) SNP and its transcription in the transcriptomics samples. The format of the seventh column is as follows: [SNP base]: [reads supporting the SNP in genomic sequencing data]: [% of total reads] ([sample name]: [SNP base]: [reads supporting the SNP in the RNA-Seq sample]: [% of total reads]). Click here for file

Additional file 5 Transcription of BWA-detected, full-length nuwts based on SNP pattern. Click here for file

Additional file 6 qPCR experiment details. Click here for file

Additional file 7 qRT-PCR experiment details. Click here for file
